# Joint Analysis of Functional and Structural Connectomes Between Preterm and Term Infant Brains via Canonical Correlation Analysis With Locality Preserving Projection

**DOI:** 10.3389/fnins.2021.724391

**Published:** 2021-10-06

**Authors:** Shu Zhang, Zhibin He, Lei Du, Yin Zhang, Sigang Yu, Ruoyang Wang, Xintao Hu, Xi Jiang, Tuo Zhang

**Affiliations:** ^1^Center for Brain and Brain-Inspired Computing Research, School of Computer Science, Northwestern Polytechnical University, Xi’an, China; ^2^School of Automation, Northwestern Polytechnical University, Xi’an, China; ^3^School of Life Sciences and Technology, University of Electronic Science and Technology of China, Chengdu, China

**Keywords:** canonical correlation analysis, locality preserving projection, human connectome, preterm and term, function and structure

## Abstract

Preterm is a worldwide problem that affects infants’ lives significantly. Moreover, the early impairment is more than limited to isolated brain regions but also to global and profound negative outcomes later, such as cognitive disorder. Therefore, seeking the differences of brain connectome between preterm and term infant brains is a vital step for understanding the developmental impairment caused by preterm. Existing studies revealed that studying the relationship between brain function and structure, and further investigating their differentiable connectomes between preterm and term infant brains is a way to comprehend and unveil the differences that occur in the preterm infant brains. Therefore, in this article, we proposed a novel canonical correlation analysis (CCA) with locality preserving projection (LPP) approach to investigate the relationship between brain functional and structural connectomes and how such a relationship differs between preterm and term infant brains. CCA is proposed to study the relationship between functional and structural connections, while LPP is adopted to identify the distinguishing features from the connections which can differentiate the preterm and term brains. After investigating the whole brain connections on a fine-scale connectome approach, we successfully identified 89 functional and 97 structural connections, which mostly contributed to differentiate preterm and term infant brains from the functional MRI (fMRI) and diffusion MRI (dMRI) of the public developing Human Connectome Project (dHCP) dataset. By further exploring those identified connections, the results innovatively revealed that the identified functional connections are short-range and within the functional network. On the contrary, the identified structural connections are usually remote connections across different functional networks. In addition, these connectome-level results show the new insights that longitudinal functional changes could deviate from longitudinal structural changes in the preterm infant brains, which help us better understand the brain-behavior changes in preterm infant brains.

## Introduction

Preterm birth is a worldwide problem that affects infants throughout their lives significantly. It is highly associated with adverse neurodevelopmental outcome in the brain and the neurodevelopmental impairment remains with the preterm birth infants which even persists into adulthood (Karolis et al., 2016). To better understand the differences caused by preterm birth in a non-invasive way, brain imaging techniques have been widely used, such as magnetic resonance imaging (MRI) (McRobbie et al., 2007), electroencephalography (EEG) (Niedermeyer and Lopes da Silva, 2005), computerized tomography (CT) (Berrington de González et al., 2009), and functional near-infrared spectroscopy (FNIRS) (Villringer and Chance, 1997). Among them, functional MRI (fMRI) and diffusion MRI (dMRI) are widely adopted to provide both reasonable temporal resolution as well as satisfying spatial resolution and tissue contrast to investigate brain function and structure (Le Bihan and Breton, 1985; Thomas, 1993; Basser et al., 2000; Sharoh et al., 2019).

Previous MRI studies have focused on either structural or functional differences between preterm and term infant brains. For example, Kashou et al. (2016) pointed out that the activation response latency is longer in preterm infants. Mahmoudzadeh et al. (2013) found that preterm infants reacted differently to voice stimuli. Similarly, a study (Saito et al., 2009) revealed that there was decreased activity in response to speech stimuli in the right temporal region and increased interhemispheric connectivity between preterm and term brains. Besides the studies above which focused on the functional perspective, tractography has also been successfully adopted in neonates to delineate major white matter tracts including the corticospinal tracts and corpus callosum (Berman et al., 2005; Aeby et al., 2009; Bassi et al., 2011; Hasegawa et al., 2011; Thompson et al., 2011). Furthermore, white matter microstructural alterations may be associated with suboptimal neurologics in certain domains at term-equivalent age (TEA) in infants born preterm (Kelly et al., 2019). However, existing studies merely focused on one single perspective (structural or functional) while the joint analysis of both functional and structural characteristics between preterm and term infant brains is very scarce. Since the brain has a specific structural substrate that provides a foundation for functional information (Calhoun, 2018), it is crucial and necessary to adopt multi-modality analysis on investigating the complex relationship between brain structure and function and further exploring such differences between preterm and term brains. In recent years, integration of both brain function and structure has been widely adopted to study the brain’s working mechanism (Groves et al., 2011; Adali et al., 2015; Calhoun and Sui, 2016) or to identify the brain alterations in diseases compared to the health controls (Calhoun et al., 2006; Arbabshirani et al., 2017; Qi et al., 2018). For example, Zhang et al. (2018a; 2018b; 2019a) proposed a framework to integrate the DICCCOL system (structural perspective) into the HAFNI system (functional perspective), and successfully obtained the consistent common landmarks with both structural and functional consistency across different individual brains. Furthermore, hierarchical representation of such multimodal representation has been proposed to show the relationship between function and structure (Zhang et al., 2019a). Kubera et al. (2019) used structure MRI and resting state fMRI to study the structure/function inter-relationships in patients with schizophrenia who have persistent auditory verbal hallucinations and achieved satisfying performance. Liu et al. (2019) proposed an advanced multimodal approach, named “Linked 4-Way Multimodal,” to identify brain differences in schizophrenia. In general, they all claimed that studying the relationship between function and structure can offer unique perspectives that may not be achieved by separated unimodal analyses. Thus, we hypothesize that joint analysis of brain function and structure can help unveil the differences between preterm and term brains.

Another important issue to explore the differences between preterm and term brains is choosing the appropriate learning algorithm. Deep learning approaches are demonstrated to be powerful and reliable for classification problems (LeCun et al., 2015; Schmidhuber, 2015). However, it always requires plenty of data to train the model adequately, which is usually a big issue in the scenario of medical imaging analysis. Independent component analysis (ICA), such as infomax (Bell and Sejnowski, 1995) and fastICA (Hyvärinen and Oja, 1997), is another widely used data-driven approach that includes two complementary constraints, independence (i.e., the maps are maximally independent of one another) and sparsity (i.e., the maps have a small number of regions with high values). Although achieving reasonable performances to study function or structure separately, ICA may not be well proposed to investigate the relationship between function and structure. To our best knowledge, canonical correlation analysis (CCA) and CCA-related approaches are most suitable methods to study the relationship among multiple profiles (Knapp, 1978; Lin et al., 2013; Zhao et al., 2017; Zhuang et al., 2017, 2020). Moreover, in order to explore the distinguishing features to differentiate the preterm and term brains, it is desirable to further modify the CCA by adding the locality preserving projection (LPP).

To explore the differences between preterm and term brains, connectome-level analysis is also important. The human connectome, which comprises many anatomically and functionally distinct regions and links by a complex network of structural white matter pathways, is widely discussed to interpret the working mechanism of the brain. As a result, connectome has been one of the fundamental ways to be adopted in the cognitive neuroscience and neuropsychology field, which is always proposed for investigating the normal brain function and disease-related dysfunction. It is worth noting that the brain connectome will significantly increase our understanding of the working mechanism of our brain, will interpret the relationship between function and structure, and will provide new insights into how brain function and structure affect each other. More importantly, examining the human brain as an integrative network of functional/structural interacting brain regions can provide a method or platform to examine how functional/structural connectivity and their interaction relates to human behavior, and how this organization may be altered during brain diseases (Sporns et al., 2005; Leergaard et al., 2012). However, many existing works merely focused on dozens of the ROIs across the whole brain, which are far from enough to model the brain’s activity globally and comprehensively (Hasegawa et al., 2011; Oishi et al., 2012; Ball et al., 2014; Jiang et al., 2015), and it will be even harder to represent the real connectivity of the whole brain. Despite the great success those works contributed, fine-scale connectome analysis is eagerly needed to interpret the working mechanism of the brain.

To tackle the abovementioned issues, in this article, we proposed a novel fine-scale CCA with LPP approach to identify the differences of brain connectome between preterm and term brains via functional and structural profiles. The advantages of the proposed method are threefold: (1) The proposed fusion framework combines both functional and structural profiles; (2) advanced CCA-LPP approach is designed to evaluate the relationship between multimodal connections, as well as consider the classification performance between preterm/term; and (3) we perform the investigation of the whole-brain differences at a finer connectome scale. The proposed method was applied to 64 subjects from Developing Human Connectome Project (dHCP) datasets (Hughes et al., 2017; Makropoulos et al., 2018), of which 32 are preterm infants and the rest are term infants. Some of our findings are in line with previous reports demonstrating that preterm/term differences can be successfully identified through our proposed framework. More importantly, our new findings that were not reported before also provide new knowledge to understand the difference of a preterm infant’s brain from term controls.

## Materials and Methods

### Overview

**FIGURE 1:**
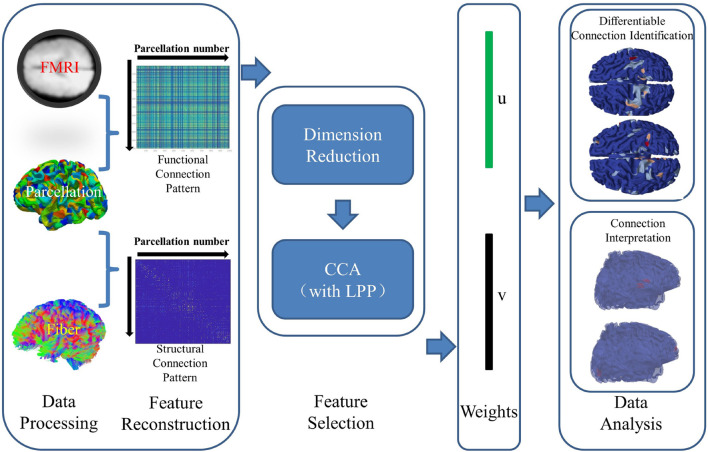
The overview of the proposed method. The major steps are summarized as follows: data preprocess, feature reconstruction, feature selection, weights, and data analysis.

The overview of the proposed framework is illustrated in Figure 1. In this work, the CCA with LPP approach is designed to identify the brain connectomes which can distinguish the preterm and term infant brains. The major steps are shown in the Figure 1.

### Data Processing

A total of 64 infant subjects were selected, including structural MRI, dMRI, and resting state fMRI (rs-fMRI) from the dHCP dataset (Hughes et al., 2017; Makropoulos et al., 2018) to perform and validate our algorithms. All individuals were scanned at TEA, which is around 40 weeks. Of them, 32 are preterm infants with birth ages ranging from 28 to 36 weeks. The others are full-term infants over 37 weeks.

For T2-weighted structural MRI, basic parameters are as follows: *TR* = 1,200 ms, *TE* = 156 ms, SENSE factor 2.11 (axial) and 2.60 (sagittal), image matrix = 290 × 290 × 203 and resolution = 0.5 mm × 0.5 mm × 0.5 mm. Diffusion weighted data consist of three shells of *b* = 400, 1,000, and 2,600 s/mm2 and interspersed with an approximately equal number of acquisitions on each shell within each run. For the rs-fMRI data, basic parameters are provided here: *TR* = 392 ms, *TE* = 38 ms, total volume = 2,300, image matrix = 67 × 67 × 45, and, resolution = 2.16 mm × 2.16 mm × 2.15 mm. The pre-processing of rs-fMRI data includes skull removal, motion correction, slice time correction, and spatial smoothing. All these steps were implemented by FMRIB Software Library (FSL) FEAT (Jenkinson et al., 2012). For the dMRI, basic parameters are provided here: *TR* = 3,800 ms, *TE* = 90 ms, total slice = 300, SENSE factor 1.2 and partial Fourier 0.86, image matrix = 128 × 128 × 64, and resolution = 1.17 mm × 1.17 mm × 1.5 mm. A spherically optimized set of directions on 4 shells (b0: 20, b400: 64, b1000: 88, b2600: 128) was split into four optimal subsets (one per phase encoding direction). DMRI was used as intra-subject standard space, to which the other data modalities are aligned. T2 weighted MRI volumes were linearly warped to FA map of dMRI. Then, the surface could be transposed to dMRI space by applying the transformation matrix onto it.

It is worth noting that cortical surfaces have been reconstructed from T2-weighted MRI data and provided in the dHCP dataset, following the steps of skull removal, tissue segmentation, and surface reconstruction. A white matter surface was then parcellated to the resolution of 200 K vertices on each hemisphere. Registration across the individual surfaces via spherical registration method and the aligned surfaces were resampled to the same number of vertices, to provide a vertex-to-vertex cross-subject correspondence. For dMRI data, skull-strip and eddy current corrections via FSL (Jenkinson et al., 2012) were applied followed by deterministic fiber tracking (6 × 10^6^ fibers in each subject) via DSI Studio (Yeh, 2020).

### Connection Reconstruction and Feature Dimension Reduction

In this article, the white matter surface was parcellated to the resolution of 500 patches of equal area on each hemisphere. Due to the cross-subject surficial alignment and resampling, the index of the patches had cross-subject correspondence as well. The cortical patches were used as graphic nodes. For functional connectome matrix, each functional connection between two nodes was weighted by the Pearson correlation coefficient of two mean fMRI signals averaged within each patch, respectively; for structural connectome matrix, each structural connection was weighed by diffusion tensor imaging (DTI) connective strength, defined as the count of DTI fibers linking the two patches.

Since the dimensions of the functional and the structural connectome matrix were as huge as 1000 × 1000, the total number of the connections was around 5 million, which would be very hard for the CCA-based approach to deal with. Thus, to reduce the dimension, in this article, we used two sample *t*-test (*p*-value < 0.05) as the constraint to exclude the connections which have least possibility to distinguish the preterm brains from term ones on both function and structure perspectives. This step was performed on function and structure matrices simultaneously, and then we selected around 5,000 common initial connections.

Then, we picked up those functional initial connections and transformed those connections with their functional connectivity features into a vector. For the functional perspective, functional initial connections with their functional connectivity features denoted by ***x*** ∈ ***R***^1×m^ (*m* = 5,000 initial connections). Similarly, for the structural perspective, structural initial connections with their structural connectivity features are also transformed into a vector, which is defined as the structural connective feature, denoted by ***y*** ∈ ***R***^1×m^. Piling the features across all subjects gives the feature matrices (***X***, ***Y*** ∈ ***R***^n×m^, n = 64) of the cohort.

### Identification of Significant Connections Through the Canonical Correlation Analysis-Locality Preserving Projection Approach

To identify the significant connections, in this work, we designed the CCA-LPP approach. CCA is to identify the maximal correlation between ***Xu*** and ***Yv*** which are linear transformations of ***X*** and ***Y***. ***X*** and ***Y*** are the functional connectivity feature matrix and structural connectivity feature matrix accordingly (size of n × m, where n is the number of subjects and m represents the number of the initial connections obtained from the last subsection). ***u*** and ***v*** are the canonical weights which provide the contribution of each feature in modeling the correlation. At the beginning, we introduced the objective function to show the whole architecture of the CCA-LPP approach. In addition, we then interpret each part of the objective function. The objective function is written as:


(1) $$\begin{array}{c} \underset{u,v}{min}-{\text{u}}^{T}{\text{X}}^{T}\text{Yv}+\lambda_{1}{||\mathrm{Xu}||}_{2}^{2}+\lambda_{2}{||\mathrm{Yv}||}_{2}^{2}+\sum_{i=1}^{2}\beta_{i}P_{i}+\sum_{i=1}^{2}\gamma_{i}R_{i}\\ +\omega_{1}{||u||}_{1}+\omega_{2}{||v||}_{1}\\ s.t.{\text{u}}^{T}{\text{X}}^{T}\text{Xu}=1,{\text{v}}^{T}{\text{Y}}^{T}\text{Yv}=1 \end{array}$$

Next, we introduce each component in detail. First, the first component and its constraints are from the conventional CCA model which is defined as follows:


(2) $$\min_{u,v}-{\text{u}}^{T}{\text{X}}^{T}\text{Yv},\text{s}.\text{t}.{\text{u}}^{T}{\text{X}}^{T}\text{Xu}=1,{\text{v}}^{T}{\text{Y}}^{T}\text{Yv}=1$$

Second, besides the relationship between brain function and structure, one of the major goals of this proposed approach is to identify the features which can differentiate the preterm and term brains. To achieve this, we adopted the LPP constraint (He and Niyogi, 2003). Specifically, two graphs ***G**_**w**_* and ***G**_**b**_* (both are size of n × n) are established to quantify the relationship between subjects. For ***G**_**w**_*, subjects within the same group are connected. For ***G**_**b**_*, subjects from different groups are connected. In detail, for ***G**_**w**_*, if subject i and subject j are in the same group:


(3) $${\text{G}}_{w}\left(i,j\right)=1,{\text{G}}_{w}\left(j,i\right)=1$$

Similarly, for ***G**_**b**_*, if subject i and subject j are in different groups:


(4) $${\text{G}}_{b}\left(i,j\right)=1,{\text{G}}_{b}\left(j,i\right)=1$$

According to ***G**_**w**_* and ***G**_**b**_*, we proposed two constraints p_1_ and p_2_ for between-group discrimination:


(5) $$\begin{array}{c} p_{1}\left(u\right)={||u||}_{D}=\alpha {\text{u}}^{\text{T}}{\text{X}}^{T}{\text{L}}_{w}\text{Xu}-\left(1-\alpha \right){\text{u}}^{T}{\text{X}}^{T}{\text{L}}_{b}\text{Xu}\\ p_{2}\left(v\right)={||v||}_{D}=\alpha {\text{v}}^{T}{\text{Y}}^{T}{\text{L}}_{w}\text{Yv}-\left(1-\alpha \right){\text{v}}^{T}{\text{Y}}^{T}{\text{L}}_{b}\text{Yv} \end{array}$$

where ***α*** is the trade-off parameter. The effect is to balance between the within-group similarity and the between-group dissimilarity. ***L*_*w*_** and ***L*_*b*_** are the Laplacian graphs of ***G*_*w*_** and ***G*_*b*_**, respectively.

For ***L**_**w**_* and ***L**_**b**_* :


(6) $${\text{L}}_{w}={\text{D}}_{w}-{\text{G}}_{w},{\text{L}}_{b}={\text{D}}_{b}-{\text{G}}_{b}$$

where ***D**_**w**_* is the degree matrix of ***G**_**w**_*, ***D**_**b**_* is the degree matrix of ***G**_**b**_*. ***L**_**w**_* and ***L**_**b**_* further represent the similarity of subjects within the same group and subjects between different groups.

Third, our hypothesis was that those preterm brains were not the same with term brains at its TEA. Thus, we regressed the features in canonical spaces {***Xu,Yv*}** to the birth ages of preterm infants to eliminate the differences and maximize their similarities. By using L2-norm to the regression, we had the following objectives:


(7) $$\begin{array}{c} R_{1}\left(u\right)={||{\text{X}}_{1}\text{u – A}||}_{2}^{2}\\ R_{2}\left(v\right)={||{\text{Y}}_{1}\text{v – A}||}_{2}^{2} \end{array}$$

where ***X***_1_, ***Y***_1_ ∈ ***R***^*n*_1_ × m^ (*n*_1_ = 32) are features from preterm group, and ***A*** ∈ ***R***^*n*_1_ × 1^ is a birth time vector, which records the birth time of all the preterm subjects.

Fourth, this objective is convex in ***u*** if we fix ***v***, and it is the same situation for ***v***. Thus, we can solve Equation 1 by the alternating iteration algorithm (Basser et al., 2000). By fixing ***u*** and ***v*** alternatively, we solved two minimization problems as follows:


(8) $$\begin{array}{c} \underset{u}{min}-{\text{u}}^{T}{\text{X}}^{T}\text{Yv}+\lambda_{1}{||\mathrm{Xu}||}_{2}^{2}+\beta_{1}P_{1}+\gamma_{1}R_{1}+\omega_{1}{||u||}_{1}\\ \underset{v}{min}-{\text{u}}^{T}{\text{X}}^{T}\text{Yv}+\lambda_{2}{||\mathrm{Yv}||}_{2}^{2}+\beta_{2}P_{2}+\gamma_{2}R_{2}+\omega_{2}{||v||}_{1} \end{array}$$

Algorithm 1 shows the implementation procedure, where we used 10^–6^ as stop criteria. It is worth noting that parameters λ, β, γ, ω are designed empirically, they are chosen to ensure: (1) the projected features yield the best classification results between preterm and term groups; (2) the projected features are strongly correlated with each other; (3) the preterm infants’ birth ages are well fitted by the projected features. λ = 0.9, β =0.8, γ = 0.8, and ω = 0.8 are suggested.


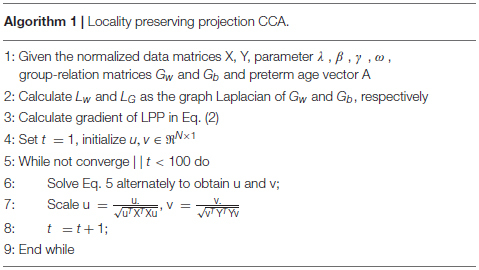


### Connectome Distance Analysis

Since local connections and global connections had huge functional and structural differences (Lu et al., 2012; Deng et al., 2014; Zhang et al., 2019b), the goal of calculating the connectome distance was to measure the distance of each connection on the cortical surface. We used *C_ij_* to denote the Euclidean distance of two patches (*i* and *j*) of a connection, and the equation of calculating the *C*_*ij*_ was shown in Equation 9. The distance *C*_*ij*_ would reveal whether the connections were anatomically close.


(9) $$\begin{array}{c} C_{ij}=\sqrt{{\left(x_{i}-x_{j}\right)}^{2}+{\left(y_{i}-y_{j}\right)}^{2}+{\left(z_{i}-z_{j}\right)}^{2}},\\ e.g.X_{i}=\left(X_{i1}+X_{i2}+\ldots +X_{in}\right)/n \end{array}$$

where *x*, *y*, *z* represent the corresponding coordinates of a patch, and each patch is consisting of *n* vertexes, the location of the patch is obtained by averaging those *n* vertexes (e.g., *i*_1_, *i*_2_, and *i*_3_).

### Comparison Experiment With Resting-State Networks

Since in Section 0, **u** and **v** were obtained and used to identify distinguishing connections. To relate these identified connections to functional networks, we chose 9 typical resting state networks (Raichle et al., 2001; Zhang et al., 2014) as the reference (please refer to Supplementary Figure 1). These brain networks were consistently observed across the subjects and tasks from fMRI scans (Lv et al., 2015a, b; Zhao et al., 2015; Zhang et al., 2016). The motivation of using 9 typical resting state networks is shown here: (1) those selected resting-state networks functionally linked brain regions that show a continuous activation during rest, e.g., DMN network and auditory network; (2) those selected resting-state networks may show an important topology that is strongly organized to their sub-functions (van den Heuvel and Hulshoff Pol, 2010). Thus, interpreting the relationship between identified connections and resting-state networks (RSN) helps to better understand the meaning of the identified connections. On this reference, we identified which two networks an identified connection links such that a 9 × 9 matrix was produced, the element of which was the count of the identified connections. Self-connection of the 9 networks, the diagonal element, was allowed.

### Comparison Experiments With Cortical Folding (Gyrus/Sulcus) Patterns

Gyri are the name given to the bump ridges on the cerebral cortex and sulci are the grooves in the cerebral cortex. Each gyrus is surrounded by sulci and vice versa. Anatomically, the gyri and sulci are existing to increase the surface area of the cerebral cortex. Many existing studies have investigated the functional/structural differences between gyri and sulci, which support us to better understand the working mechanism of our brain. For example, Deng et al. (2014) proposed that gyri are more likely to be the functional connection centers, which are responsible for exchanging information among remote structurally connected gyri and nearby sulci; on the contrary, sulci exchange information directly with their nearby gyri. Zhang et al. (2019b) proposed similar conclusions from a different perspective, they found that gyri are more global functional integration centers while sulci are more local processing units, they further reveal that gyri tend to be simpler lower frequency signal components while sulci are more complex higher frequency signal components. Hence, it is important and valuable to study the associated cortical folding patterns on the two ends of those identified connections, which will help us to better understand functional meanings of those identified connections.

We related the identified connections with their associated cortical folding patterns. Since the connection links two cortical patches, we determined the folding patterns of the patches. A patch was labeled as “gyrus” or “sulcus” by its mean curvatures of the vertexes of the patch (>0.5 as gyrus and <0.5 as sulcus). By this way, a connection can be classified as a “Gyrus-Gyrus” connection, a “Gyrus-Sulcus” one, or a “Sulcus-Sulcus” one. Every connection would be measured and given one class label.

## Results

### Identified Connections via Canonical Correlation Analysis With Locality Preserving Projection

**FIGURE 2:**
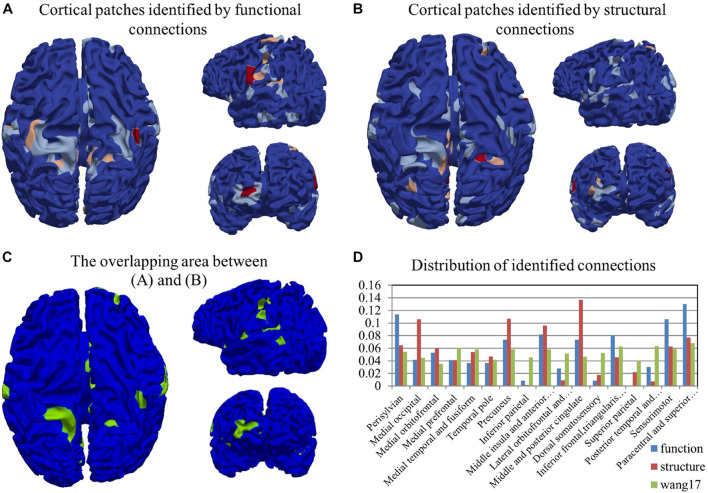
The visualization of cortical patches linked by the identified functional connections **(A)** and structural connections **(B)**. The color-bar from blue to red represents the indices of cortical patches. **(C)** The overlapping area between **(A,B)**. **(D)** The distribution of identified functional and structural connections. The distribution of the whole brain cortical surface is also provided as a reference in green.

By adopting the proposed CCA with LPP approach, 89 functional connections and 97 structural connections were identified in total. We highlighted those patches linked by these connections in Figures 2A,B and denoted them by activation areas. In these figures, we can clearly see that some patches are connected more than once, and they are colored red or pink. Activation areas in Figures 2A,B were found on the parietal lobe, the temporal lobe, and the occipital lobe from the identified functional connections. The results revealed that the parietal lobe was an especially important region to differentiate preterm and term infants, which is in line with existing studies (Saito et al., 2009; Mahmoudzadeh et al., 2013; Kashou et al., 2016). Besides, parts of the temporal lobe and the occipital lobe could also be found that were activated. These results were also consistent with many existing preterm studies (Saito et al., 2009; Mahmoudzadeh et al., 2013; Kashou et al., 2016). By comparison between the results of Figures 2A,B, it revealed that there are large portions of areas on which both function and structure are co-activated (visualized in Figure 2C). The overlap between functional (Figure 2A) and structural (Figure 2B) activation area is shown in Figure 2C. The Jaccard similarity and overlap size was about 29.38%, because some patches were connected more than once. If we took the total activation times of each patch into consideration, the overlap rate was rising to 35.64%, which represented the relatively close relationship between structure and function. To better locate the activation areas, we used a state-of-the-art infant brain atlas “Wang17” (Li et al., 2015; Wu et al., 2019) as a reference. The identified functional/structural connections and the activation areas were reported and quantified in this atlas (Figure 2D). According to Figure 2D, activation areas of function and structure are not the same, and the discrepancy is further confirmed by using the atlas in Figure 2D as the reference. The distribution of comparison results clearly shows that the functional and structural activation areas are quite different in certain areas. In detail, in areas of “Perisylvian,” “Inferior frontal, triangularis, and opercularis,” “Sensorimotor,” and “Paracentral and superior frontal” the function shows a large possibility of activation; in areas of “Medial occipital,” “Precuneus,” and “Middle and posterior cingulate,” the structure performs with a much higher chance to be active.

Furthermore, we investigated the characteristics of these identified connections between preterm and term groups. On one hand, for the functional perspective, we paid attention on the Pearson correlation of two corresponding patches. Identified functional connections in the term group had significantly higher average weights than those in the preterm group in which 86 out of 89 connections were term-dominant connections, and only 3 were preterm-dominant ones. It was suggested that term brains had more functional similarity on those identified functional areas. On the other hand, the intensity of structural connections was the number of the fibers that connected the corresponding two patches. After the statistical analysis, among the 97 connections, 68 of them were preterm-dominant connections, which means preterm has a stronger structural intensity than the term one. The left 29 connections were term-dominant connections. These results suggested that preterm infants have more fiber connections on those identified structural areas.

### Identified Connections and Their Dispersion Characteristics

In addition to the overlap between functional and structural activated areas in Figures 2A,B, the differences between them can be observed as well. Regarding their spatial distribution, structural activation areas were relatively more dispersed than functional activation areas.

**FIGURE 3:**
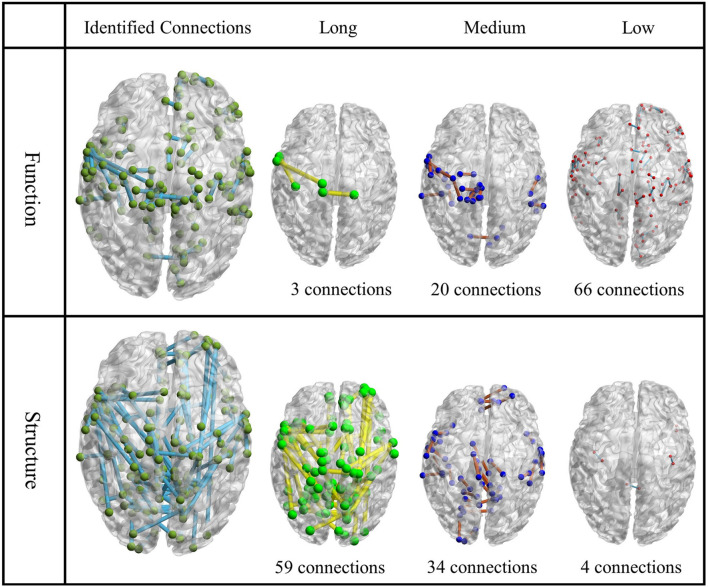
The visualization of identified functional connections and structural connections. The first column is the identified functional/structural connections; the other three columns are their subdivided levels. Each node represents a patch, and the location of the node is the center of that patch.

To better quantify this disparity between them, we classified the connection between two activation areas to low connection (distance ≤10), medium connection (10 < distance ≤20), and long connection (distance > 20). Figure 3 showed the identified connection on structural and functional matrices, respectively, as well as those classified into the three length groups (Xia et al., 2013). To quantitatively, for the functional connections, the total number of connections in each length group (from low to long) is 66, 20, and 3, respectively; for the structural connections, the number is 4, 34, and 59, respectively. In addition, the average distance of functional connections is 9.52 ± 5.19 (mean ± STD, same to the rest), while the average distance of structural connections is 26.07 ± 14.93, which is almost three times larger than the former ones.

**FIGURE 4:**
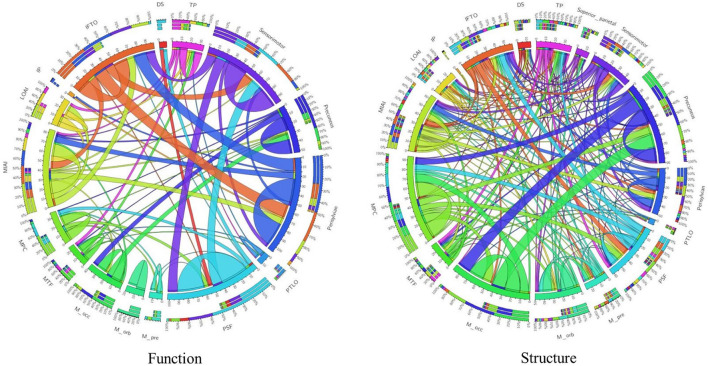
Visualization of connection patterns using Wang17 atlas as a reference. Functional connection map and structural connection map are provided, respectively. There are 17 areas from Wang17 atlas that are visualized in the wheel chart as seeds. To be simple, abbreviations of areas from Wang17 atlas are used in the charts. Full names are summarized in the Supplementary Table 1.

By using “Wang17” functional atlas as the reference, we illustrated the identified connections as well as their connective patterns among the 17 functional areas in Figure 4. The components from functional connections were relatively simple, and a large portion of connections were found within the functional area, such as “Perisylvian” and “Medial orbitofrontal,” while the connections between networks were relatively less. On the contrary, structural connections were relatively complex, with abundance of connections between networks. To quantitatively, on one hand, the intensity of intra-area functional connections is 10.7 ± 9.0, while the intensity of intra-area structural connections is 5.2 ± 5.8, the intensity of intra-area functional connections is obviously larger than structural connections and the *p*-value of significant test of them is obtained as 0.0405. On the other hand, the intensity of inter-area functional connections is 0.72 ± 2.15, while the intensity of inter-area structural connections is 1.07 ± 1.87, we can see that for the inter-area connections, structural connections are dominant, and the *p*-value of the significant test of them is recorded as 0.0507.

Besides, the inter-hemispheric connections and intra-hemispheric connections are two fundamental connection types, which represent different meanings. van den Heuvel et al. (2015) proposed that the majority of all large-scale pathways from preterm brains (both intra- as well as interhemispheric tracts) tend to be present at term, together with an adult-like small-world modular architecture. There are some pathways (both intra- as well as interhemispheric tracts) that are distinguished between preterm and term brains, which is supported in our findings. To our best knowledge, there are few studies further analyzing those that are distinguished as intra- as well as interhemispheric tracts between preterm and term brains. Thus, our findings show some new insights for further understanding the development of neonatal connectome. In this work, among the selected 89 functional connections, 4 (4.49%) functional connections are observed in the interhemispheric type, and 85 (95.51%) are obtained as the intra-hemispheric ones. Among the selected 97 structural connections, 27 (27.84%) and 70 (76.21%) structural connections are identified with interhemispheric and intra-hemispheric connections, respectively. Therefore, it is clearly shown that structural changes have stronger possibilities linking two hemispheres.

### Relationship Between Functional/Structural Connections and Resting-State Networks

**FIGURE 5:**
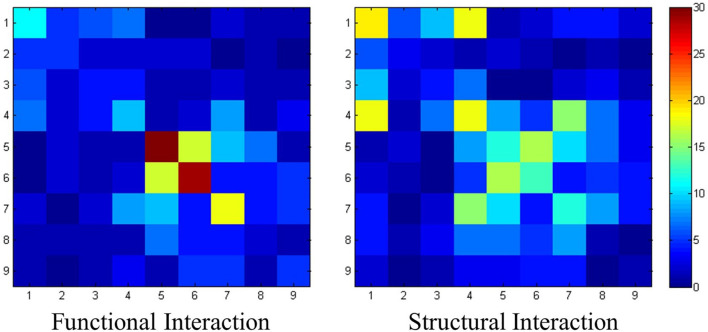
The interaction pattern of functional connections and structural connections. Nine resting-state networks are utilized here to statistically analyze the identified connections. The value of each element represents the number of the connection patches located within the corresponding resting-state networks.

We further investigated whether the distribution of the connections identified in section “Identified Connections and Their Dispersion Characteristics” was related to their concurrency with resting-state functional networks. The assumption was that long connections may have a higher chance to be present between these functional networks, while local connections are most likely to connect the regions within a network. The relation between connections and resting-state functional networks is shown in Figure 5. To quantitatively, the intensity of intra-network functional connections is 13.2 ± 12.0, while the intensity of intra-network structural connections is 9.2 ± 7.1. However, the intensity of inter-network functional connections is 3.53 ± 3.38, while the intensity of inter-network structural connections is 5.32 ± 4.47.

The results show that more structural connections are observed between networks, due to heavier connections distributed off the diagonal. On the contrary, connections with high intensities could be observed on the diagonal in the functional connection matrix. Especially for network #5 and network #6, the intensities of intra-network functional connections are 36 and 29, respectively, which are the highest ones in the network.

### Differences of Connectomes Between Functional and Structural Connections With Their Associated Cortical Folding Patterns

**FIGURE 6:**
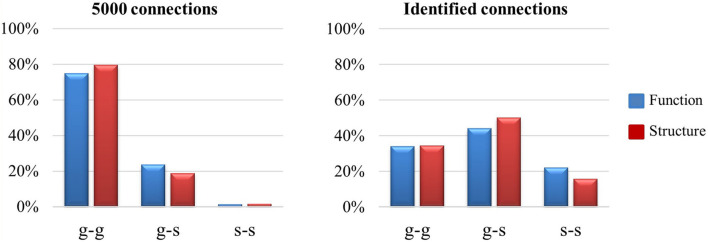
The visualization of gyrus/sulcus distributions between *t*-test connections and identified connections. There are 5000 connections (*p* < 0.05) counted for all the connections after the dimension reduction (*t*-test) step in section “Connection Reconstruction and Feature Dimension Reduction,” identified connections are the connections identified after the CCA-LPP approach.

We further investigated the relation between cortical folding patterns and the identified connections. We quantified the folding patterns as gyrus or sulcus, of the patches linked by connections. In our previous studies (Lu et al., 2012; Deng et al., 2014), gyral regions were linked by more long-range structural and functional connections while sulcal regions were linked by more local connections. These results were reproduced by our experiment, and it is shown in the left panel of Figure 6. In the left panel of Figure 6, 5000 connections are utilized, which were selected after the step of dimension reduction (*t*-test) in section “Connection Reconstruction and Feature Dimension Reduction.” The “g-g” connections are obviously dominant, which occupied around 80% connections, “g-s” and “s-s” are relatively weak, which owned nearly 20% and 1%, respectively. However, in the right panel of Figure 6, the results reveal that g-s and s-s connections accounted for a larger proportion when compared with the left panel which suggests that “g-s” and “s-s” connections have more possibility to be altered on preterm infant brains.

In addition, we compared the connection patterns between function (blue bar) and structure (red bar) on selection connections. The results in the right panel of Figure 6 support our findings in section “Identified Connections and Their Dispersion Characteristics,” where the identified functional connections include more s-s connections while identified structural connections include more g-s connections. In other words, identified functional connections have more local connections than the identified structural connections.

## Discussion and Conclusion

In this article, we proposed a novel strategy to integrate function and structure profiles together and to investigate the connectome differences between preterm and term brains. In total, 89 functional connections and 97 structural connections were identified through our CCA-LPP approach. “Perisylvian,” “Inferior frontal, triangularis and opercularis,” “Sensorimotor,” “Paracentral and superior frontal areas,” “Medial occipital,” “Precuneus,” and “Middle and posterior cingulate” were the key regions that we needed to pay more attention to for investigating the differences between preterm and term brains. By further investigating the characteristics of those identified functional/structural connections, we found that the identified differentiable functional connections were local path connections, while those differentiable structural connections were long path connections. Further analysis based on the RSN and gyrus/sulcus patterns confirmed our hypothesis that functional differences were usually found within the typical brain networks and especially for the sulci-sulci connections, but structural differences were usually found linking the different typical brain networks, thus less sulci-sulci connections were found when compared with functional connections, but more gyrus/sulcus connections were observed.

We want to emphasize that multi-modality analysis is adopted in this study to investigate both differentiable structural and functional brain connectomes between the preterm and term infants. To achieve this, we design a novel CCA with the LPP approach. In this approach, two major parts are mentioned. One is CCA; the CCA part is adopted to integrate brain function and structure together and study their relationship. Another one is LPP; the LPP part is proposed to improve the ability to differentiate the preterm and term brains. Thus, the goal of proposing this approach is efficiently discovering the differentiable connections from both functional and structural perspectives.

It is worth noting many studies have pointed out that brain networks have been developed at the preterm stage. And it is a particularly important finding, which supports our studies to investigate the relationship between identified connections and brain networks. Kashou et al. (2016) claimed that hallmark or generational structures of the human connectome are present before term birth and subject to early development. To evaluate this finding, we also adopted the online dictionary learning and sparse coding algorithm (Mairal et al., 2009; Lv et al., 2015b) to discover the brain networks from each preterm/term infant brain images. After we checked the brain components obtained from the ODL algorithm, the results substantiated the hypothesis. Typical functional brain networks include typical resting state networks that can be observed both in the preterm and term infants (Please refer to Supplementary Material section “Online Dictionary Learning and Sparse Coding Algorithm” and “Functional Networks Obtained From ODL Method”).

Our findings reveal that identified differentiable functional connections are always short path connections; however, identified differentiable structural connections are always long path connections. To our best knowledge, it is the first time this phenomenon was discovered. In previous studies, it is well known that there is a structural–functional coupling theory (van den Heuvel et al., 2015; Wang et al., 2015). van den Heuvel et al. (2015) proposed that a positive SC-FC coupling was found to be present in all neonates, which suggests that higher levels of structural connectivity are associated with higher levels of functional coupling. In this work, we agree that function and structure are supporting each other. This phenomenon can also be seen from the identified differentiable areas in Figure 1 from both the functional perspective and the structural perspective, they are quite similar (the overlap rate is about 30%). However, when we work on the connection level, clearly differences are still observed between function and structure (only one connection is both selected from functional and structural profiles), so we believe that even though we always claim that function and structure are supporting each other, they are two modalities fundamentally and they play special roles for the working mechanism of the brain. In addition, we assume that in the stage of preterm brain development, the way of functional and structural connectome changes may be different, structural connectome changes maybe more related with long distance connections, and functional connectome changes possibly focus more on local connections, this will be further confirmed in the longitudinal studies in our future work. Anyway, we argue that multi-modality is necessary for better understanding how the brain works.

Many studies have devoted much effort to explore the differences of development between preterm and term brains (Villringer and Chance, 1997; Niedermeyer and Lopes da Silva, 2005; Berrington de González et al., 2009; Sharoh et al., 2019). In conclusion, for the preterm infants, the risk of cerebral palsy, visual impairment, and learning abilities are increased. They may lead to health issues and cognitive function problems. For the motor performance, the motor impairment might be associated with cerebral hypoxia during the transition immediately to birth in preterm infants (Berman et al., 2005; Bassi et al., 2011; Hasegawa et al., 2011; Thompson et al., 2011). For the speech perception, language disorder is a major concern, because preterm infants were exposed to auditory stimuli in the form of utterances and preterm infants follow different development traces due to differences in intrauterine and extrauterine development (Boardman et al., 2006, 2010). Similarly, for the facial recognition, injury of right temporal lobe, prefrontal lobe and fusiform gyrus may lead to the impairment of facial recognition function by the extrauterine environment (Kapellou et al., 2006; van Kooij et al., 2012; van den Heuvel et al., 2015). In general, we can see that extrauterine to the environment, transition birth, and exposure to the stimulus are the main reasons for causing the preterm/term differences.

For the future work, first, we plan to detect the differentiable connections through longitudinal studies. Longitudinal studies can assist us to describe the change of our brain via the timelines. It will be much easier for us to identify the vital changes and observe their tiny changes; second, we would like to introduce multi-modality CCA analysis, which means we want to introduce more modalities into our model. Besides function connectome and structure connectome, there are other brain modalities, such as thickness, cortical folding patterns, as well as other anatomical information. We believe that more modalities can bring more complementary information for better investigation results. In this way, brain changes can be revealed and elaborated through brain connectome, and further analysis can provide instructions for clinical diagnosis and therapy.

## Data Availability Statement

The original contributions presented in the study are included in the article/Supplementary Material, further inquiries can be directed to the corresponding author/s.

## Author Contributions

SZ, ZH, and TZ: conception and design. SZ, ZH, XJ, and TZ: analysis and interpretation. YZ and XH: data collection. SZ, XJ, and TZ writing the manuscript. SZ, XJ, SY, and TZ: critical revision of the manuscript. SZ, ZH, and RW: statistical analysis. SZ and TZ: overall responsibility. All authors contributed to the article and approved the submitted version.

## Conflict of Interest

The authors declare that the research was conducted in the absence of any commercial or financial relationships that could be construed as a potential conflict of interest.

## Publisher’s Note

All claims expressed in this article are solely those of the authors and do not necessarily represent those of their affiliated organizations, or those of the publisher, the editors and the reviewers. Any product that may be evaluated in this article, or claim that may be made by its manufacturer, is not guaranteed or endorsed by the publisher.
