# Supplementary material for: Joint Analysis of Functional and Structural Connectomes Between Preterm and Term Infant Brains via Canonical Correlation Analysis With Locality Preserving Projection
Source: Front Neurosci. 2021 Oct 6;15:724391. doi: 10.3389/fnins.2021.724391 (PMC8526737; doi:10.3389/fnins.2021.724391)
Supplement: Supplementary file 1 [file Presentation_1.PDF]

Supplementary Material

1 Supplementary Method

1.1 Visualization of typical resting-state networks

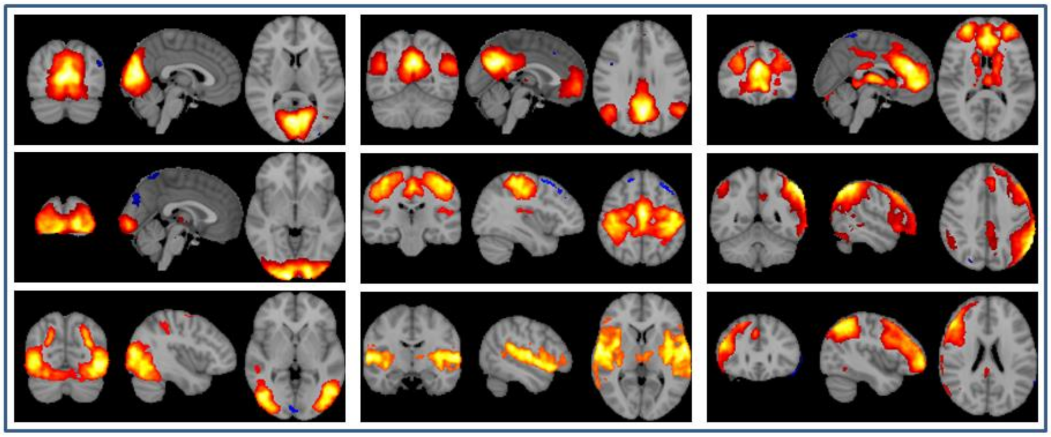

Supplementary Figure 1. The visualization of nine typical RSN networks.

1.2 Online dictionary learning and sparse coding algorithm

Individual functional networks are obtained via online dictionary learning and sparse coding algorithm (ODL) (Mairal et al., 2009). It was adopted to learn a dictionary with sparsity constraint from the whole-brain fMRI signal matrix $\boldsymbol{S}$ (with time length t and voxel number n). To represent $\boldsymbol{S}$, the algorithm would decompose a dictionary $\boldsymbol{D}$ consisting of k atoms with the corresponding sparse loading coefficient matrix α. Set $\boldsymbol{S} = [s_1, s_2, s_3 ... s_n] \in \mathbb{R}^{t*n}$ Thus, each signal from S can be reconstructed by a combination of dictionary atoms. Loss function $\ell(S, D)$ is defined in Eq. (1).

$$l(S, D) \triangleq min_{\alpha^i \in \mathbb{R}} \frac{1}{2} \|S - D\alpha\|_F + \lambda \|\alpha\|_{1,\ 1} \qquad (1)$$

where λ is a regularization parameter to trade off the regression residual and sparsity level. Each column in dictionary $\boldsymbol{D}$ represents a typical temporal signal from $\boldsymbol{S}$ and each row in coefficient matrix α can be mapped back onto the brain volume to show its spatial distribution patterns, termed functional component. In this work, 200 components are eventually obtained from $\boldsymbol{S}$ for each subject.

2 Supplementary Result

2.1 Functional networks obtained from ODL method

By adopting the online dictionary learning algorithm, 200 functional components are obtained for each subject. Then Eq. (2) is applied to search the functional components which are highly overlapped with 9 RSN templates.

$$O_{(A,B)} = \frac{2 * (A \cap B)}{(A \cup B)} \quad (2)$$

Where A and B represent two brain networks, O is overlap between A and B. We randomly chosen two subjects and visualize the matching results in Fig.2. In general, most RSN templates are found to be highly related to components from every subject, and the average overlap rate (Dice coefficient) is 0.38 ± 0.13, suggesting that our identified functional components include the common resting state networks. This is in line with previous reports that most functional networks are already observed both in preterm and term birth (Mahmoudzadeh et al., 2013; Saito et al., 2009; Aeby et al., 2009), which demonstrating the effectiveness of the online dictionary learning method as well as the point that brain networks are already developed during the preterm stage.

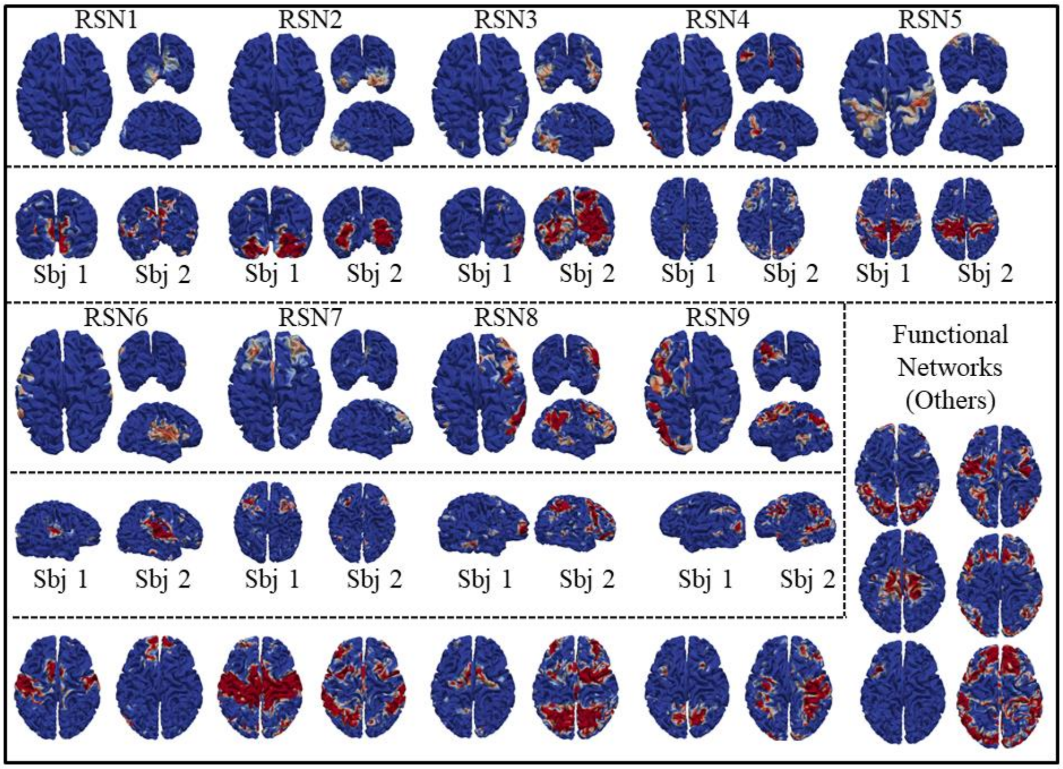

Supplementary Figure 2. Obtained functional networks from ODL algorithm. Two subjects are randomly chosen from our dataset as examples.

2.2 Abbreviation of the Wang17 template

Supplementary Table 1. Abbreviation of the Wang17 template

Area Name	Abbreviation
Perisylvian	(No Abbreviation)
Medial occipital	M_occ
Medial orbitofrontal	M_orb
Medial prefrontal	M_pre
Medial temporal and fusiform	MTF
Temporal pole	TP
Precuneus	(No Abbreviation)
Inferior parietal	IP
Middle insula and anterior insula	MIAI
Lateral orbitofrontal and anterior insula	LOAI
Middle and posterior cingulate	MPC
Dorsal somatosensory	DS
Inferior frontal, triangularis and opercularis	IFTO
Superior parietal	SP
Posterior temporal and lateral occipital	PTLO
Sensorimotor	(No Abbreviation)
Paracentral and superior frontal	PSF
